# Uncovering the Top Nonadvertising Weight Loss Websites on Google: A Data-Mining Approach

**DOI:** 10.2196/51701

**Published:** 2024-12-11

**Authors:** Carlos A Almenara, Hayriye Gulec

**Affiliations:** 1 School of Health Sciences Universidad Peruana de Ciencias Aplicadas Lima Peru; 2 Interdisciplinary Research Team on Internet and Society Faculty of Social Studies Masaryk University Brno Czech Republic

**Keywords:** consumer health informatics, cyberattack risk, data mining, Google, information seeking, weight loss, online health information, website analysis, digital health, internet search

## Abstract

**Background:**

Online weight loss information is commonly sought by internet users, and it may impact their health decisions and behaviors. Previous studies examined a limited number of Google search queries and relied on manual approaches to retrieve online weight loss websites.

**Objective:**

This study aimed to identify and describe the characteristics of the top weight loss websites on Google.

**Methods:**

This study gathered 432 Google search queries collected from Google autocomplete suggestions, “People Also Ask” featured questions, and Google Trends data. A data-mining software tool was developed to retrieve the search results automatically, setting English and the United States as the default criteria for language and location, respectively. Domain classification and evaluation technologies were used to categorize the websites according to their content and determine their risk of cyberattack. In addition, the top 5 most frequent websites in nonadvertising (ie, nonsponsored) search results were inspected for quality.

**Results:**

The results revealed that the top 5 nonadvertising websites were healthline.com, webmd.com, verywellfit.com, mayoclinic.org, and womenshealthmag.com. All provided accuracy statements and author credentials. The domain categorization taxonomy yielded a total of 101 unique categories. After grouping the websites that appeared less than 5 times, the most frequent categories involved “Health” (104/623, 16.69%), “Personal Pages and Blogs” (91/623, 14.61%), “Nutrition and Diet” (48/623, 7.7%), and “Exercise” (34/623, 5.46%). The risk of being a victim of a cyberattack was low.

**Conclusions:**

The findings suggested that while quality information is accessible, users may still encounter less reliable content among various online resources. Therefore, better tools and methods are needed to guide users toward trustworthy weight loss information.

## Introduction

Seeking online health information is prevalent among Americans [1,2]. In 2013, a representative survey of adult internet users in the United States found that 27% had looked online for information about losing or controlling their weight in the last 12 months [3]. General search engines are the first tool for health-related queries [4]. With an average of over 80 billion monthly visits in 2022, Google is the most preferred search engine for such internet-based activity [5].

Although limited, the available evidence points to a lack of quality and comprehensiveness regarding weight loss information in English and Spanish on websites in the United States and the German language web [6-8]. It also indicates the potential risk of accessing information with doubtful credibility, such as deceiving advertisements about commercial and fad diets [9]. These findings are concerning because online health information impacts health decisions and behaviors [3]. Furthermore, low-quality or misleading information might be detrimental for those who have eating disorder-related symptomatology or obesity because previous research identified a higher frequency for the use of weight loss websites among individuals with body image and body weight concerns [10].

Google Trends, an open-source repository, is a popular tool for retrieving the most common search queries about weight loss in previous studies [11-14]. Yet, the Google search engine offers additional “Autocomplete Suggestions” and “People Also Ask” features based on users’ queries. To our knowledge, no previous study used search queries that combined the data from Google Trends and the additional features of the Google Search engine to determine the most frequently accessed weight loss websites. This study aimed to identify and describe the characteristics of the top weight loss websites on Google using an automated approach that combined data from Google Trends and the additional features of the Google Search engine.

## Methods

### Data Mining

On April 6, 2021, a comprehensive list of 432 unique search queries about weight loss (Multimedia Appendix 1) was retrieved from the Answer Socrates database [15] with the search term “weight loss” (without quotations). Answer Socrates is a database of users’ search queries combining Google search suggestions (Autocomplete Suggestions), “People Also Ask” featured questions and Google Trends data. We developed a data-mining software tool (Multimedia Appendix 2) to search for each query in Google and automatically retrieve the results. The tool sets the United States as the default geographical location and English as the default language. In this study, we set the United States as the default location because it is the largest English-speaking country. According to Google Trends, the third largest volume of “weight loss” searches since 2004 has occurred in the United States, after Trinidad and Tobago and South Africa [16]. The tool was developed with Python (Python Software Foundation) programming language and designed to gather Google Search engine results through an application programming interface (API) developed by SerpApi [17]. More precisely, it is a Python-code tool for data mining that automatically searches each query on Google and retrieves the results for each query in a JSON file. Each JSON file contains Google advertising results, “Google Places” results, “People Also Ask” results, and organic search results, including web address, title, and description. Organic results are nonadvertising (ie, nonsponsored) search results that appear because they are relevant to the search terms [18]. By contrast, nonorganic or sponsored or advertising results are paid advertisements and appear above organic results. The Python code tool creates a database (a CSV file) that includes the top 100 organic results for each query (Multimedia Appendix 3). To test for the validity of this software tool, the first author performed a manual verification. The tool was set to Peru as the default geographical location because the first author was located in Peru. English was set as the default language because 10 queries were randomly selected from the total of 432 English queries used in this study. A manual search in Google was done separately for each of those 10 queries to compare the results obtained with the software tool. The manual searches were done with an anonymous web browser (Mozilla Firefox) in Peru, with English as the default language of the search engine. The key data (ie, title, description, URL) of the first 10 nonadvertising results for each query were compared, and no differences were found. The tool was used on April 14, 2021. This study focused solely on organic search results. This decision was based on the fact that advertising or sponsored results have already been studied, although several years ago [6], and they are idiosyncratic because they are regulated by algorithms. In other words, advertising results are tailored to each user and, therefore, do not represent the results retrieved for all users, as is the case for organic results.

### Data Analyses

**Figure 1:**
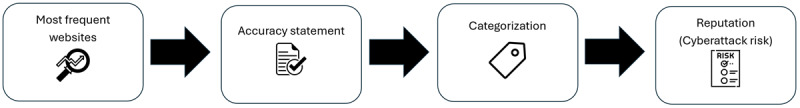
Steps of the data analysis procedure.

The data analysis procedure is represented in the flowchart below (Figure 1).

First, the most frequent websites for the top 5 Google Search results were calculated using Python code (Multimedia Appendix 2). This study focused on the top 5 organic search results because nearly 90% of the clicks occur on Google’s first 5 nonadvertising results [7]. This clicking behavior might suggest users’ tendency to perceive the information presented at the top as most relevant and helpful. Since we focused on queries looking for answers about weight loss on websites, we expected a similar clicking behavior in this study. Next, the 5 most frequent websites were manually inspected to evaluate their accuracy. Like previous studies [6], we examined whether the websites presented an “accuracy statement” that indicated that the provided information was evidence-based, fact-checked, or, at least, had been reviewed to ensure its trustworthiness. Any statement like this within the website indicated that the website provided an accuracy statement. The websites were also examined to evaluate whether author credentials were disclosed when weight loss information was provided. When the authorship of the weight loss information was given, the website was regarded as providing author credentials.

Then, all the unique websites that appeared in the top 5 results were categorized. There are several methods to categorize websites, including manual and automated processes. A recent study examined several domain classification services and provided directions for future research using website categories⁠ [19]. The study found that manual categorization of websites tends to be biased due to disagreements resulting from subjective opinions. In addition, the inherent ambiguity of many categories in taxonomy and the dual nature of many websites contribute to this bias [19]. For example, taxonomies containing thousands of categories and websites with ambiguous content, such as tourism blogs advertising casinos, make it difficult to categorize them manually. On the other hand, the study found that domain classification services vary in coverage, meaning that some websites are categorized because the service indexes them, whereas others do not. Furthermore, the accuracy of the categorization is affected by inconsistent taxonomies. Finally, there is low agreement among these services. Consequently, the authors suggest that researchers can manually examine random subsets of categorizations to determine if the labeling quality is sufficient for the purpose of the research study and rely on classification services with category labels acquired through a well-documented process and incorporated into a thoroughly vetted taxonomy [19]. Based on those results and recommendations, the following domain classification services were tested: BrightCloud, Curlie, Cyren, FortiGuard, McAfee, VirusTotal, and Zvelo. After manually inspecting the results, we found that Zvelo [20] produced the most meaningful and accurate classification labels with a reasonable number of categories (nearly 500).

Zvelo is a domain classification service with more than 13 years of service. The categories are generated by Zvelo’s human-supervised artificial intelligence (AI) models, and they currently use fourth-generation AI models (personal communication, January 31, 2023). For each URL (ie, web address), the Zvelo database provides 3 classification groups, each with up to 3 categories [20]. The first group is the primary classification provided by Zvelo; this is the one used in this study. The other two are additional categorizations provided by the Interactive Advertising Bureau (IAB), a marketing-orientated taxonomy [21]. Consistent with the recommendations made by Vallina et al [19], Zvelo was preferred over the IAB taxonomy because it provided more extensive coverage of the websites in the dataset. The manual inspection of the website categories revealed that Zvelo’s category labels were also more relevant to the content than the IAB’s. This observation is probably because the IAB is a marketing-oriented domain classification service. We also preferred the Zvelo classification due to its state-of-the-art AI technology use. All categories for each URL are available in Multimedia Appendix 4. The categories were retrieved between January 2023 and February 2023.

Finally, each URL was given a reputation score based on Webroot’s BrightCloud IP Reputation Services [22]. The reputation score predicts the risk that an IP will deliver a cyberattack (ie, it identifies malicious addresses). Scores are obtained using big data and a machine-learning algorithm. Scores are grouped into 5 categories: High-risk (1-20), Suspicious (21-40), Moderate-risk (41-60), Low-risk (61-80), and Trustworthy (81-100) [19].

### Ethical Considerations

Given that no human participants participated in this study and the data were public, the study was deemed exempt from the institutional review board approval process.

## Results

After applying the Python-code tool to each JSON file, a CSV file was created containing a database with the 432 search queries and the Top 100 organic search results (Multimedia Appendix 3).

Table 1 shows the count of the most frequent website results, the trustworthiness, the reputation, and the web categorization of the top 5 website results.

**Table 1:** The 5 most frequent websites within the top 5 Google search results (N=2160).

Website	Total^a^, n (%)	Accuracy statement	Author credentials	Reputation (0-100)^b^	Category
healthline.com	197 (9.12)	Yes	Yes	96	Health
webmd.com	91 (4.21)	Yes	Yes	96	Health
verywellfit.com	85 (3.94)	Yes	Yes	88	Nutrition & diet
mayoclinic.org	79 (3.66)	Yes	Yes	100	Health
womenshealthmag.com	61 (2.82)	Yes	Yes	88	Women’s health
Other	1647 (76.25)	—^c^	—	—	—
Total	2160 (100)	—	—	—	—

^a^Total number of times the website appeared in the top 5 results.

^b^Cyberattack risk. Scores higher than 80 indicate a trustworthy website.

^c^Not applicable.

There were 2160 results (the first 5 organic search results multiplied by 432 queries) and 623 unique websites (Multimedia Appendix 5). The most frequent website was healthline.com, which appeared 197/2160 (9.12%) times in the top 5 organic search results. This was followed by webmed.com, verywellfit.com, mayoclinic.org, and womenshealthmag.com. These top 5 most frequent websites provided accuracy statements (eg, fact-check statements) and author credentials. The authors of healthline.com, webmd.com, and verywellfit.com were usually health care professionals. In the case of mayoclinic.org, the authorship was usually declared as “Mayo Clinic Staff.” womenshealthmag.com had several authors who were described as “freelance writers.” In addition, healthline.com, verywellfit.com, and womenshealthmag.com explicitly provided a fact-check statement to assure users that the information was evidence-based and written by health care professionals.

**Figure 2:**
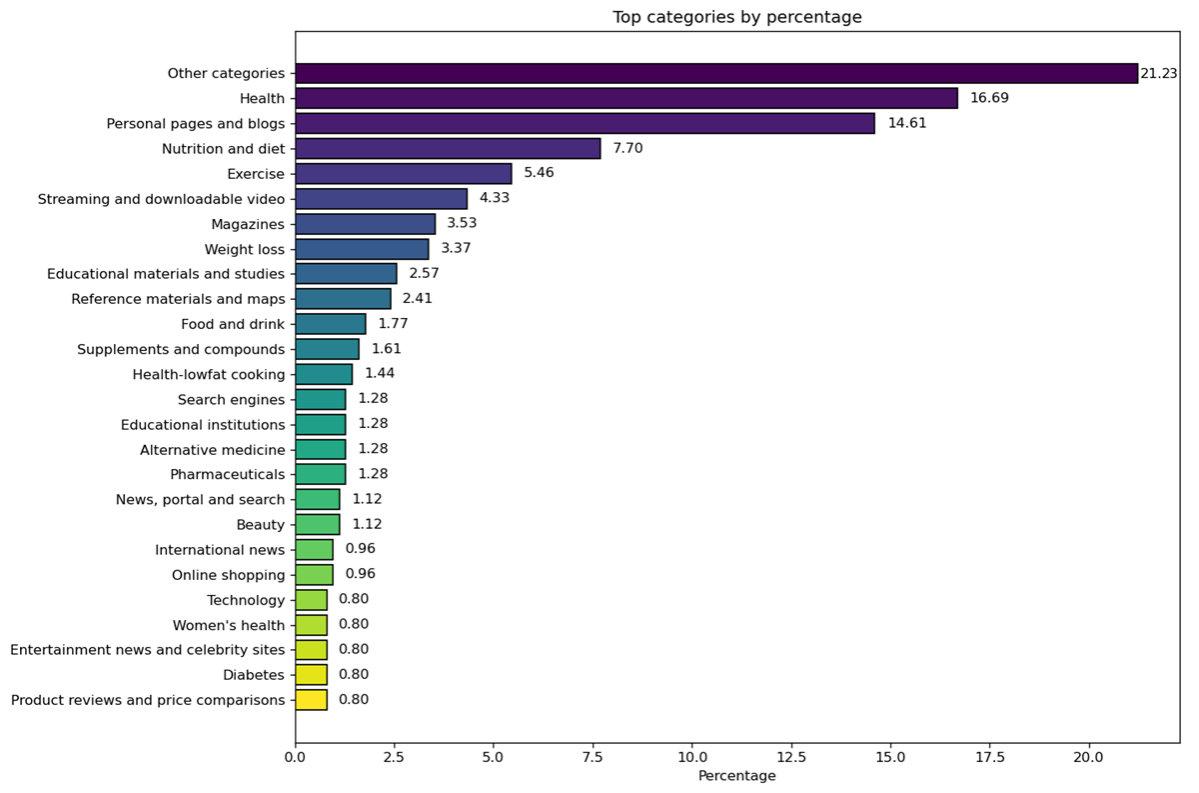
Frequency and percentage of website categories.

Next, the 623 websites were categorized by the Zvelo categorization taxonomy [23]. In total, 101 unique categories were found (Multimedia Appendix 4). The categories with less than 5 appearances were grouped into “Other Categories.” As can be seen in Figure 2, most websites appeared less than 5 times (Other categories: 132/623 times, 21.23%), indicating a large diversity of results.

The following most common categories were “Health” (104/623, 16.69%) and “Personal Pages and Blogs” (91/623, 14.61%), followed far behind by “Nutrition and Diet” (48/623, 7.7%) and “Exercise” (34/623, 5.46%). The most common websites in the “Health” category tended to ensure that they provided the most reliable information (eg, healthline.com). By contrast, “Personal Pages and Blogs” had little to no information about the authorship and accuracy of the information provided, although with some exemptions (eg, thehealthy.com).

Table 2 shows the 5 most frequent websites for the most frequent categories (not including the “Other Categories”).

**Table 2:** Examples of the most frequent websites for the top categories.

	Health	Personal pages and blogs	Nutrition and diet	Exercise
1	healthline.com	simple-nourished-living.com	verywellfit.com	shape.com
2	webmd.com	obesitycoverage.com	livestrong.com	muscleandfitness.com
3	mayoclinic.org	thehealthy.com	mediweightloss.com	core-trainingpt.com
4	medicalnewstoday.com	developgoodhabits.com	precisionnutrition.com	sparkpeople.com
5	prevention.com	blog.myfitnesspal.com	trifectanutrition.com	thinwithin.com

With some exemptions (4.01%), most websites ranked 79 or higher (95.99%) in reputation, with a mean reputation score of 89.85 (SD 8.53). This finding indicates that the average reputation score of websites was trustworthy (Multimedia Appendix 4). The reputation scores for the 5 most frequent websites within the top 5 Google search results are displayed in Table 1**.**

## Discussion

### Principal Findings

This study identified and described the characteristics of the top weight loss websites on Google using an automated approach that combined data from Google Trends and the additional features of the Google Search engine. The 5 most frequent websites for weight loss were healthline.com, webmd.com, verywellfit.com, mayoclinic.org, and womenshealthmag.com. They provided fact-checked or evidence-based information about weight loss, written by health care professionals with disclosed credentials. We used the Zvelo domain classification service to categorize the search results. The results yielded a wide diversity of website categories. The most common category was “Other Categories,” accounting for around one-fifth of all websites. It was followed by “Health,” “Personal Pages and Blogs,” “Nutrition and Diet,” and “Exercise” categories, which altogether accounted for 45% (277/623) of the search results. When we examined the most frequent 5 websites under these categories, only the “Health” category involved websites with fact-checked or evidence-based weight loss information that disclosed the author’s credentials. By comparison, the information on authorship and the accuracy of the statements was mainly lacking in the “Personal Pages and Blogs,” “Nutrition and Diet,” and “Exercise” categories. Finally, we found that the average reputation of all websites was trustworthy, which indicates that there is no security risk and users are very unlikely to be victims of cyberattacks by these weight loss websites.

### Comparison With Previous Work

Previous studies found that websites with higher quality and comprehensiveness rank low in the search results [6]. Although we identified that evidence-based and fact-checked information was conveyed by the most frequent websites in the top 5 organic search results, it should be noted that what users retrieve as a result of their search queries may not always lead them to the websites with credible information, given that they represent only a fraction of the total number of websites. Furthermore, websites with quality content may get lost among the myriad results, including advertising results that appear first on websites like Google.

Previous studies adopted the standard website nomenclature and manual categorization of websites based on their source (eg, commercial sites, medical sites, government sites, university sites, news sites, online media sites, and blogs) [6-8]. In this study, we used the Zvelo domain classification service, which uses an AI-based classification engine to apply a topic-based taxonomy for URL classification. Given that we applied this approach for the first time on weight loss websites, there is no previous evidence to compare our findings. Nevertheless, it is noteworthy that earlier studies reported nutrition and exercise as the most frequently addressed topics across the website categories on weight loss. We found that “Personal Pages and Blogs” was a frequent category in the search results, which means that individuals who seek online weight loss information might frequently encounter personal accounts and histories on English-language weight loss websites in the United States. Given that the most frequent websites under “Personal Pages and Blogs,” “Nutrition and Diet,” and “Exercise” categories have suboptimal quality, users who endorse these websites may be at risk of inadequate and even misleading information.

### Future Directions

In this study, we evaluated the 5 most frequent websites under each category and determined whether they disclosed author credentials and an accuracy statement to ensure their quality. As a next step, future studies can examine each topic category based on other quality criteria, such as the frequency and scope of the evidence-based information provided using manual or automated approaches. This information might give a more in-depth understanding of the content and quality of the website categories. For instance, websites in the “Health” category might be more trustworthy because they integrate a more holistic approach by addressing several evidence-based domains (eg, healthy eating, nutrition, diet, exercise, pharmacotherapy, and behavioral change strategies) for successful weight loss.

Cyberattacks have social and psychological consequences, such as the series of emotional reactions that can follow a cyberattack (feeling violated, powerless, angry, rage, grief, shame, etc), and previous research has highlighted the importance of addressing them [24]. Thus, there are diverse opportunities for future studies, such as studying how cyberattacks target weight loss advertisements and the psychological consequences of these attacks for individuals looking to lose weight.

### Strengths

Previous studies generated search queries with convenience samples for the terms with which people would search the internet for weight loss information [6-8]. The search terms were then categorized and submitted to the Google Trends repository to obtain the final search queries. The total number of search queries ranged between 26 and 30. In this study, we retrieved 432 unique search queries from Answer Socrates [15], which combined Google search suggestions (Autocomplete suggestions), “People Also Ask” featured questions and Google Trends data. We also used a data-mining software tool to determine the top 5 most frequent websites that appeared among the 5 organic search results. This study provided initial evidence that a data-mining software tool can help identify the most common websites for weight loss. This automated approach might also help to inspect other health-related search queries submitted to the Google search engine.

### Limitations

Although this study has strengths, such as the large number of search queries used and the automated approach to retrieve search results and categorize websites, it has some limitations. First, our results are concerned with English-language websites found in the United States; thus, they cannot be generalized to other locations. Further research may focus on different locations for English search queries related to weight loss and compare the Google search results across locations. Second, notwithstanding the fact that actual Google users make the search queries, it is unclear how Google selects them. Therefore, future studies could use different approaches, such as an ecological design, to gather search queries when users search the internet for weight loss information. For example, real-time data capture techniques, including an ecological momentary assessment, can be used in a large sample to study the behavior of individuals while looking for weight loss information [25]. Such a design also has the advantage of tracking various concurrent details, such as the time of day, the season, the geographical location, sociodemographics, anthropometrics, and current mood, to study their association with the search terms. Similarly, case study research can be used to explore this phenomenon because it allows for an in-depth exploration of individual behaviors, contextual influences, and the factors that shape the information-seeking process [26]. This approach would help develop a comprehensive understanding of how individuals interact with weight loss information online, including the challenges they face and the strategies they use to navigate the abundance of available online information.

Third, we used a data-driven and AI-based domain classification approach using Zvelo. Nevertheless, the categories produced by Zvelo were not readily interpretable. There was a broad diversity of website categories and possible overlaps between them (eg, “Nutrition & Diet,” “Health-Low Fat Cooking,” “Food & Drink”). The interpretability of AI-based domain classification should be determined in future studies. Fourth, we manually inspected websites to find the accuracy statements (eg, fact-check statements). These statements were usually in sections like “About Us.” Unfortunately, this method is time-consuming, so future studies can look for a way to automate this task so that all the websites can be examined for accuracy statements. For example, a third-party authenticator can provide a certificate to websites that provide accurate information about weight loss and an API to developers so they can easily access this information.

Fifth, from the data-mining results, it was clear that sponsored results (ie, advertising), which were not included in the analyses, were assigned the top results in Google searches. Weight loss advertising already has many deceiving pieces of information, with hundreds of cases presented to the Federal Trade Commission⁠ [27,28]. In addition, Google’s online advertising is regulated by algorithms that consider idiosyncratic information about the internet user, such as sociodemographic information, so the ads are better tailored to the user. Future studies could evaluate how this information is displayed to the user, how users interact with it, and how they use it in their everyday lives, particularly in their attempts to lose weight, to evaluate the risks and benefits of interacting with online advertising results. These studies could use an automated approach like the one used in this study.

Therefore, initiatives should be taken to improve web results so that users can get reliable information about weight loss. For example, a web browser plugin that identifies the top health websites can mark them with an icon so that users can quickly identify them. An example of such a web browser plugin is Web of Trust [29], which provides a website reputation rating, and it marks URLs with a green (trusted), yellow (suspicious), red (untrusted), or gray (unknown) icon.

Furthermore, governments can create policies to ensure websites disclose how they handle misinformation and fact-checking. Websites can also be required to label content that has been fact-checked, along with the source of the fact-check. Weight loss information generated by AI, such as ChatGPT, should also be disclosed. Internet users should look up websites for the “accuracy statements” indicating that the weight loss information is “evidence-based,” “fact-checked,” or at least “reviewed by” to ensure its trustworthiness. Similarly, consumers should inspect whether author credentials are disclosed when weight loss information is provided, such as the name of the person who wrote the information, their profession, academic degrees, and links to further professional information (eg, LinkedIn profile page). Software developers can design algorithms to down-rank or flag potentially misleading content or design web browser plugins to alert users interacting with misinformation.

### Conclusions

We developed a data-mining approach to identify and evaluate the top nonadvertising weight loss websites found through Google searches and analyzed 432 search queries. The 5 most frequent websites were healthline.com, webmd.com, verywellfit.com, mayoclinic.org, and womenshealthmag.com. These sites consistently provided fact-checked, evidence-based information with author credentials, indicating high trustworthiness. We also classified websites into various categories using an automated approach and found “Health,” “Personal Pages and Blogs,” “Nutrition and Diet,” and “Exercise” as the most common categories. The findings suggested that while quality information is accessible, users may still encounter less reliable content among various online resources. Therefore, better tools and methods are needed to guide users toward trustworthy weight loss information.

## Data Availability

The datasets generated during and/or analyzed during this study are available in the Open Science Framework repository [30].

## Appendix

Multimedia Appendix 1 Queries.

Multimedia Appendix 2 Python code.

Multimedia Appendix 3 Results.

Multimedia Appendix 4 Categories.

Multimedia Appendix 5 Google weight loss total count of top 5.

## Abbreviations

AI: artificial intelligence
API: application programming interface
IAB: Interactive Advertising Bureau
