# Supplementary material for: Uncovering the Top Nonadvertising Weight Loss Websites on Google: A Data-Mining Approach
Source: JMIR Infodemiology. 2024 Dec 11;4:e51701. doi: 10.2196/51701 (PMC11669867; doi:10.2196/51701)
Supplement: Multimedia Appendix 2 [file infodemiology_v4i1e51701_app2.pdf]

Uncovering the top non-advertising weight loss websites on Google: A data mining approach

Part 1 - Scraping Google search results

Authors: Carlos A. Almenara (https://almenara.io) & Hayriye Gulec

Date: April 2021

In [1]:
```
import os
import time
import pandas as pd
import json
from serpapi import GoogleSearch # serpAPI documentation: https://serpapi.co
```

In [2]:
```
# Set working directory
os.chdir('/home/user/google_weight_loss')
```

In [3]:
```
# Load serpAPI key:
with open('serp_api_key', 'rb') as f:
    api_key = f.readline().rstrip()
    f.close
```

In [4]:
```
# Loading the dataset of 432 queries from answersocrates.com (the column 'id
with open('weightloss_queries.csv', 'rb') as w:
    queries = pd.read_csv(w, index_col='id')
    w.close
queries.head()
```

Out[4]:

id	type	modifier	question
1	questions	Are	are weight loss pills good
2	questions	Are	are weight loss patches safe
3	questions	Are	are weight loss clinics worth it
4	questions	Are	are weight loss programs covered by insurance
5	questions	Are	are weight loss dna tests accurate

In [5]:
```
# How many unique questions do we have
queries['question'].nunique()
```

Out[5]: 432

In [5]:

```
# Querying and exporting each result as a JSON file
start = time.time()
for i in queries['question']:
    params = {
      "engine": "google",
      "api_key": api_key,
      "q": i,
      "location": "585069bfee19ad271e9bc673", # id for United States
      "device": "desktop",
      "hl": "en",
      "gl": "us",
      "num": "100", # 0 to 99
      "async": "false",
      "output": "json"
    }
    search = GoogleSearch(params)
    dict_results = search.get_dict()
    file_name = i + '.txt'
    with open(file_name, 'w') as outfile:
        json.dump(dict_results, outfile)
        outfile.close
end = time.time()
print('Finished after ' + str(end - start) + ' seconds.')
```

```
Finished after 2100.1502029895782 seconds.
```

In [ ]:

```
Uncovering the top non-advertising weight loss websites on Google: A data
mining approach

Part 2 - Creating a dataframe with all link results

Authors: Carlos A. Almenara (https://almenara.io) & Hayriye Gulec

Date: April 2021
```

In [1]:
```
import os
import time
import pandas as pd
import json
```

In [2]:
```
# Set working directory
os.chdir('/home/user/google_weight_loss/results')
```

In [3]:
```
# Make a list of files in folder WITH file extension
path = '/home/user/google_weight_loss/results'
list1 = os.listdir(path)
print(list1[:10])
```

```
['what weight loss pills actually work.txt', 'weight loss for men.txt', 'ar
e weight loss programs covered by insurance.txt', 'weight loss is hard.tx
t', 'weight loss breakfast ideas.txt', 'weight loss lifting plan.txt', 'wei
ght loss after baby.txt', 'why weight loss is so slow.txt', 'weight loss pi
lls compared to phentermine.txt', 'where weight loss shows first.txt']
```

In [4]:
```
# Make a list of files in folder WITH and WITHOUT file extension
list2 = [os.path.splitext(filename)[0] for filename in os.listdir(path)]
print(list2[:10])
```

```
['what weight loss pills actually work', 'weight loss for men', 'are weight
loss programs covered by insurance', 'weight loss is hard', 'weight loss br
eakfast ideas', 'weight loss lifting plan', 'weight loss after baby', 'why
weight loss is so slow', 'weight loss pills compared to phentermine', 'wher
e weight loss shows first']
```

In [5]:
```python
# Let's create a list of lists for all link results
data = []
start = time.time()
for i in list1:
    with open(i, 'rb') as f:
        query = json.load(f)
        results = query['organic_results']
        links = [ val['link'] for val in results ]
        data.append(links)
        f.close
end = time.time()
print('Finished after ' + str(round(end - start, 2)) + ' seconds.')
```

```
Finished after 0.15 seconds.
```

In [6]:
```python
# Creating a dataframe: columns are queries, rows are link results
df = pd.DataFrame(data)
df = df.transpose()
df.columns = list2
df.head()
```

Out[6]:

	what weight loss pills actually work	weight loss for men	
**0**	https://www.healthline.com/nutrition/12-weight...	https://www.menshealth.com/weight-loss/a195368...	
**1**	https://observer.com/2020/11/best-weight-loss-...	https://www.healthline.com/nutrition/weight-lo...	https
**2**	https://www.discovermagazine.com/sponsored/bes...	https://www.mensjournal.com/health-fitness/50-...	htt
**3**	https://www.medicalnewstoday.com/articles/320646	https://www.fitfatherproject.com/weight-loss-t...	https
**4**	https://www.niddk.nih.gov/health-information/w...	https://www.lifehack.org/articles/lifestyle/li...	

5 rows × 432 columns

In [7]:
```python
# Write dataframe into csv file
with open('google_weight_loss_results.csv', 'w') as w:
    df.to_csv(w)
    w.close
```

```
Uncovering the top non-advertising weight loss websites on Google: A data
mining approach

Part 3 - Parsing URLs to get the top level domain

Authors: Carlos A. Almenara (https://almenara.io) & Hayriye Gulec

Date: April 2021
```

In [1]:
```python
import os
import pandas as pd
from urllib.parse import urlparse
```

In [2]:
```python
# Set working directory
os.chdir('/home/user/Documents')
```

In [3]:
```python
# Loading the dataset of 432 queries and their respective link results
with open('google_weight_loss_results.csv', 'r', encoding='utf-8') as f:
    dataset = pd.read_csv(f, index_col=0)
    f.close
dataset.head()
```

Out[3]:

	what weight loss pills actually work	weight loss for men	
**0**	https://www.healthline.com/nutrition/12-weight...	https://www.menshealth.com/weight-loss/a195368...	
**1**	https://observer.com/2020/11/best-weight-loss-...	https://www.healthline.com/nutrition/weight-lo...	https
**2**	https://www.discovermagazine.com/sponsored/bes...	https://www.mensjournal.com/health-fitness/50-...	htt
**3**	https://www.medicalnewstoday.com/articles/320646	https://www.fitfatherproject.com/weight-loss-t...	https
**4**	https://www.niddk.nih.gov/health-information/w...	https://www.lifehack.org/articles/lifestyle/li...	

5 rows × 432 columns

In [4]:
```python
# Subsetting the first 5 results
df = dataset.iloc[:5]
df.head()
```

Out[4]:

	what weight loss pills actually work	weight loss for men	
0	https://www.healthline.com/nutrition/12-weight...	https://www.menshealth.com/weight-loss/a195368...	
1	https://observer.com/2020/11/best-weight-loss-...	https://www.healthline.com/nutrition/weight-lo...	https
2	https://www.discovermagazine.com/sponsored/bes...	https://www.mensjournal.com/health-fitness/50-...	htt
3	https://www.medicalnewstoday.com/articles/320646	https://www.fitfatherproject.com/weight-loss-t...	https
4	https://www.niddk.nih.gov/health-information/w...	https://www.lifehack.org/articles/lifestyle/li...	

5 rows × 432 columns

In [6]:
```python
# A list of our queries
queries = df.columns.tolist()
print(queries[:5])
```

```
['what weight loss pills actually work', 'weight loss for men', 'are weight
loss programs covered by insurance', 'weight loss is hard', 'weight loss br
eakfast ideas']
```

In [7]:

```
# Replacing URLs with top level domain
for i in queries:
    for l in df[i]:
        sub_domain = urlparse(l).netloc
        top_domain = '.'.join(sub_domain.split('.')[-3:])
        df[i] = df[i].replace(l, top_domain)
# NOTE: there are some caveats of using this method, see: https://pandas.pyd
df.head()
```

```
/tmp/ipykernel_6912/2424813018.py:6: SettingWithCopyWarning:
A value is trying to be set on a copy of a slice from a DataFrame.
Try using .loc[row_indexer,col_indexer] = value instead

See the caveats in the documentation: https://pandas.pydata.org/pandas-doc
s/stable/user_guide/indexing.html#returning-a-view-versus-a-copy (https://p
andas.pydata.org/pandas-docs/stable/user_guide/indexing.html#returning-a-vi
ew-versus-a-copy)
  df[i] = df[i].replace(l, top_domain)
```

Out[7]:

	what weight loss pills actually work	weight loss for men	are weight loss programs covered by insurance	weight loss is har
**0**	www.healthline.com	www.menshealth.com	blogs.webmd.com	www.everydayhealth.coı
**1**	observer.com	www.healthline.com	quotewizard.com	www.verywellfit.coı
**2**	www.discovermagazine.com	www.mensjournal.com	pocketsense.com	www.cnet.coı
**3**	www.medicalnewstoday.com	www.fitfatherproject.com	www.healthline.com	www.npr.oı
**4**	niddk.nih.gov	www.lifehack.org	www.netquote.com	rightasrain.uwmedicine.oı

5 rows × 432 columns

In [9]:
```
# Replace www.
df2 = df.replace('www.','', regex=True)
df2.head()
```

Out[9]:

	what weight loss pills actually work	weight loss for men	are weight loss programs covered by insurance	weight loss is hard	weight
**0**	healthline.com	menshealth.com	blogs.webmd.com	everydayhealth.com	
**1**	observer.com	healthline.com	quotewizard.com	verywellfit.com	
**2**	discovermagazine.com	mensjournal.com	pocketsense.com	cnet.com	womensl
**3**	medicalnewstoday.com	fitfatherproject.com	healthline.com	npr.org	
**4**	niddk.nih.gov	lifehack.org	netquote.com	rightasrain.uwmedicine.org	

5 rows × 432 columns

In [10]:
```
# Is there any missing value?
nan = df2.isnull().values.any()
print(nan)
```

```
False
```

In [10]:
```
# Write dataframe into csv file
with open('google_weight_loss_results_topdomain_new_test.csv', 'w') as w:
    df2.to_csv(w)
    w.close
```

In [ ]:

```
Uncovering the top non-advertising weight loss websites on Google: A data
mining approach

Part 4 - Parsing URLs to get the top level domain

Authors: Carlos A. Almenara (https://almenara.io) & Hayriye Gulec

Date: April 2021
```

In [3]:
```
import os
import pandas as pd
from urllib.parse import urlparse
from  collections import Counter
```

In [4]:
```
# Set working directory
os.chdir('/home/user/Documents')
```

In [5]:
```
# Loading the dataset of 432 queries and their respective link results (top
with open('google_weight_loss_results_topdomain_new_test.csv', 'r', encoding
    dataset = pd.read_csv(f, index_col=0)
    f.close
dataset.head()
```

Out[5]:

	what weight loss pills actually work	weight loss for men	are weight loss programs covered by insurance	weight loss is hard	weight
**0**	healthline.com	menshealth.com	blogs.webmd.com	everydayhealth.com	
**1**	observer.com	healthline.com	quotewizard.com	verywellfit.com	
**2**	discovermagazine.com	mensjournal.com	pocketsense.com	cnet.com	womensl
**3**	medicalnewstoday.com	fitfatherproject.com	healthline.com	npr.org	
**4**	niddk.nih.gov	lifehack.org	netquote.com	rightasrain.uwmedicine.org	

5 rows × 432 columns

In [6]:
```
# Is there any missing value?
nan = dataset.isnull().values.any()
print(nan)
```

```
False
```

In [7]:
```python
# Subsetting the first 10 results
df1 = dataset.iloc[:10]
# Transposing
df = df1.transpose()
print(df.shape)
df.head()
```

(432, 10)

Out[7]:

	0	1	2	3	
**what weight loss pills actually work**	healthline.com	observer.com	discovermagazine.com	medicalnewstoday.com	
**weight loss for men**	menshealth.com	healthline.com	mensjournal.com	fitfatherproject.com	
**are weight loss programs covered by insurance**	blogs.webmd.com	quotewizard.com	pocketsense.com	healthline.com	
**weight loss is hard**	everydayhealth.com	verywellfit.com	cnet.com	npr.org	rig
**weight loss breakfast ideas**	eatthis.com	healthline.com	womenshealthmag.com	eatingwell.com	

In [ ]:
```python
# Write dataframe into csv file
with open('google_weight_loss_transposed.csv', 'w') as w:
    df.to_csv(w)
    w.close
```

```
Uncovering the top non-sponsored weight loss websites on Google: A data
mining approach

Part 5 - Parsing URLs to get the top level domain

Authors: Carlos A. Almenara (https://almenara.io) & Hayriye Gulec

Date: April 2021
```

In [1]:
```
import os
import pandas as pd
```

In [2]:
```
# Set working directory
os.chdir('/home/user/Documents')
```

In [3]:
```
# Loading the dataset of 432 queries and their respective link results (top
with open('google_weight_loss_transposed_top5.csv', 'r', encoding='utf-8') a
    dataset = pd.read_csv(f, index_col=0)
    f.close
dataset.head()
```

Out[3]:

	0	1	2	3	
**what weight loss pills actually work**	healthline.com	observer.com	discovermagazine.com	medicalnewstoday.com	
**weight loss for men**	menshealth.com	healthline.com	mensjournal.com	fitfatherproject.com	
**are weight loss programs covered by insurance**	blogs.webmd.com	quotewizard.com	pocketsense.com	healthline.com	
**weight loss is hard**	everydayhealth.com	verywellfit.com	cnet.com	npr.org	rig
**weight loss breakfast ideas**	eatthis.com	healthline.com	womenshealthmag.com	eatingwell.com	

In [5]:
```
dataset.rename(columns={'0': 'Rank_01', '1': 'Rank_02', '2': 'Rank_03', '3':
                        '4': 'Rank_05', '5': 'Rank_06', '6': 'Rank_07', '7':
                        '8': 'Rank_09', '9': 'Rank_10'}, inplace=True)
```

In [ ]:
```
"""
# # Most common websites in RANK_01 with Percent column
df1 = pd.DataFrame(columns=['Count', 'Percent'])
df1['Count'] = dataset.Rank_01.value_counts()[:10]
list = []
for i in df1['Count'].values:
    perc = round(i*100/432, 2)
    list.append(perc)
df1['Percent'] = list
df1[:10]
"""
```

In [8]:
```
# Most common websites in RANK_01
s1 = dataset.groupby('Rank_01').size()
df1 = s1.to_frame(name='Count')
df1 = df1.sort_values(by='Count', ascending=False, na_position='first')
df1[:10]
```

Out[8]:

	Count
**Rank_01**	
**healthline.com**	74
**webmd.com**	30
**womenshealthmag.com**	18
**verywellfit.com**	16
**mayoclinic.org**	14
**amazon.com**	9
**simple-nourished-living.com**	7
**prevention.com**	7
**goodhousekeeping.com**	6
**eatthis.com**	6

In [9]:
```
# Most common websites in RANK_02
s2 = dataset.groupby('Rank_02').size()
df2 = s2.to_frame(name='Count')
df2 = df2.sort_values(by='Count', ascending=False, na_position='first')
df2[:10]
```

Out[9]:

	Count
**Rank_02**	
**healthline.com**	65
**webmd.com**	18
**mayoclinic.org**	15
**verywellfit.com**	15
**eatthis.com**	12
**pinterest.com**	10
**womenshealthmag.com**	10
**yelp.com**	9
**prevention.com**	8
**amazon.com**	6

In [10]:
```
# Most common websites in RANK_03
s3 = dataset.groupby('Rank_03').size()
df3 = s3.to_frame(name='Count')
df3 = df3.sort_values(by='Count', ascending=False, na_position='first')
df3[:10]
```

Out[10]:

	Count
**Rank_03**	
**healthline.com**	23
**mayoclinic.org**	21
**verywellfit.com**	21
**webmd.com**	16
**womenshealthmag.com**	13
**medicalnewstoday.com**	10
**amazon.com**	7
**pinterest.com**	7
**shape.com**	7
**eatthis.com**	6

In [11]:
```
# Most common websites in RANK_04
s4 = dataset.groupby('Rank_04').size()
df4 = s4.to_frame(name='Count')
df4 = df4.sort_values(by='Count', ascending=False, na_position='first')
df4[:10]
```

Out[11]:

	Count
**Rank_04**	
**healthline.com**	20
**mayoclinic.org**	19
**verywellfit.com**	13
**womenshealthmag.com**	12
**webmd.com**	12
**medicalnewstoday.com**	12
**eatingwell.com**	9
**eatthis.com**	9
**prevention.com**	8
**livestrong.com**	7

In [12]:
```
# Most common websites in RANK_05
s5 = dataset.groupby('Rank_05').size()
df5 = s5.to_frame(name='Count')
df5 = df5.sort_values(by='Count', ascending=False, na_position='first')
df5[:10]
```

Out[12]:

	Count
**Rank_05**	
**verywellfit.com**	20
**healthline.com**	15
**webmd.com**	15
**mayoclinic.org**	10
**prevention.com**	9
**medicalnewstoday.com**	9
**womenshealthmag.com**	8
**today.com**	8
**eatthis.com**	7
**everydayhealth.com**	7

In [13]:
```
# Transformind dataframes into dictionaries to later merge
dic1 = dict(df1.iloc[:,-1])
dic2 = dict(df2.iloc[:,-1])
dic3 = dict(df3.iloc[:,-1])
dic4 = dict(df4.iloc[:,-1])
dic5 = dict(df5.iloc[:,-1])
```

In [14]:
```
# Merging dic1 and dic2
result_1 = {key: dic1.get(key,0) + dic2.get(key,0) for key in set(dic1) | se

# Merging result_1 and dic3
result_2 = {key: result_1.get(key,0) + dic3.get(key,0) for key in set(result

# Merging result_2 and dic4
result_3 = {key: result_2.get(key,0) + dic4.get(key,0) for key in set(result

# Merging result_3 and dic5
result_4 = {key: result_3.get(key,0) + dic5.get(key,0) for key in set(result
```

In [15]:
```
# Creating dataframe from merged dictionaries
new = pd.DataFrame(result_4, index=['Total'])
new = new.transpose()
```

In [16]:
```
# Write dataframe into csv file
with open('google_weight_loss_TOTAL_top_5_final.csv', 'w') as w:
    new.to_csv(w)
    w.close
```
